# Establishment of a Murine Chronic Anorexia Nervosa Model

**DOI:** 10.3390/cells12131710

**Published:** 2023-06-24

**Authors:** Anna Staffeld, Sadaf Gill, Annelie Zimmermann, Natalie Böge, Katharina Schuster, Stephan Lang, Markus Kipp, Rupert Palme, Linda Frintrop

**Affiliations:** 1 Institute of Anatomy, Rostock University Medical Center, 18057 Rostock, Germany; 2 Unit of Physiology, Pathophysiology and Experimental Endocrinology, Department of Biomedical Sciences, University of Veterinary Medicine Vienna, A-1210 Vienna, Austria

**Keywords:** anorexia nervosa, activity-based anorexia, running wheel activity, amenorrhea, brain volume

## Abstract

Anorexia nervosa (AN) is associated with hyperactivity, amenorrhea, and brain atrophy. The underlying pathophysiology is mostly unknown, and new targets for therapeutic interventions are needed. This study aimed to systematically establish a murine AN model with the parameter extent of starvation, animal age, and length of starvation for functional studies. The activity-based anorexia (ABA) model combines food restriction with running wheel access. Early adolescent and adolescent mice received 40% of their baseline food intake until a 20% or 25% weight reduction was reached (acute starvation). To mimic chronic starvation, body weight loss was maintained for another two weeks. Running activity was examined using wheel sensors, while amenorrhea was investigated by analysis of vaginal smears. Brain sections were used to analyze cerebral cortex volumes. Acute starvation did not lead to either AN-related symptoms, whereas chronic starvation led to hyperactivity and amenorrhea except in the adolescent cohort with 20% weight reduction. Only ABA mice with 25% weight reduction revealed a cortex volume reduction. The optimal parameters to mirror AN-related symptoms included a 25% weight reduction, early adolescent or adolescent mice, and chronic starvation. The ABA model enables functional analysis of the impact of chronic AN on the underlying hormonal, behavioral, and brain pathophysiology.

## 1. Introduction

Anorexia nervosa (AN) has a particular significance among eating disorders due to its high prevalence and mortality rates [1]. This mental health disorder is characterized by an intense fear of gaining weight, distorted body image, and restrictive eating behavior that leads to significant weight loss. In addition, the hallmarks of this disorder include hyperactivity, amenorrhea, and hypercortisolism [2]. Neuropathological studies have found severe brain volume changes, including reductions in white and grey matter [3]. Although the brain volume loss of AN patients is related to the extent of neuropsychological deficits [4,5,6], the underlying pathophysiology is mostly unknown. Therefore, animal models are urgently needed to find new targets for therapeutic interventions, due to the limited effectiveness of current treatment concepts for AN.

The most widely used animal model is the activity-based anorexia (ABA) model, which combines food restriction with access to a running wheel (reviewed in [7,8,9]). The original ABA model consists of 2–3 h restricted food access per day leading to an increase in locomotor activity [10]. This model mimics core somatic symptoms of AN such as body weight loss, hyperactivity, amenorrhea, and endocrinological alterations associated with an activation of the hypothalamic-pituitary-adrenal (HPA) axis [11,12,13,14]. The original animal model had a higher risk of mortality because some animals became extremely active during feeding periods. To address this issue, we modified the ABA model in rats slightly by providing the animals with only 40% of their daily food intake [15,16]. This modification helped to avoid the increased mortality risk of the original model while allowing the investigation of chronic starvation.

Currently, there is no established procedure for the modified ABA model in mice. Thus, the aim was to establish this model in mice systematically for functional studies, considering the following parameters: (i) extent of body weight reduction (20 vs. 25%), (ii) age of the animals (early adolescent vs. adolescent), and (iii) length of starvation (acute vs. chronic starvation). The primary indicators to establish the model were hyperactivity, amenorrhea, and brain atrophy, with the latter estimated via the cerebral cortex volume. A secondary aim of our study was to analyze the welfare of the animals by investigating stress behavior and the HPA axis. In addition, blood glucose levels after acute and chronic starvation were investigated to get insights about the energy metabolism of the ABA mice. Specifically, we analyzed the impact of starvation on nestling behavior and fecal corticosterone metabolites as well as on blood glucose levels.

## 2. Materials and Methods

### 2.1. Animals

The C57BL/6J mice (4 weeks old (4W), early adolescent, and 8 weeks old (8W), adolescent) were female only and obtained from Janvier Labs (Le Genest-Saint-Isle, France). Given the higher prevalence of AN in female compared with male patients [17,18,19,20], our study focused on female mice. This also allowed us to use amenorrhea as an AN-related symptom representing an adequate level of starvation. In total, 120 mice were housed under a 12/12 h light/dark cycle at a temperature of 22 ± 2 °C. The beginning of the light phase was at 6 AM and the beginning of the dark phase was at 6 PM. Furthermore, the cages were changed weekly and microbiological monitoring was included as advised by the Federation of European Laboratory Animal Science Associations (FELASA) recommendations. The animal investigations were approved by the Review Boards for the Care of Animal Subjects of the district government of Mecklenburg-Western Pomerania (reference number 7221.3-1-005/21).

### 2.2. Study Design

**Figure 1:**
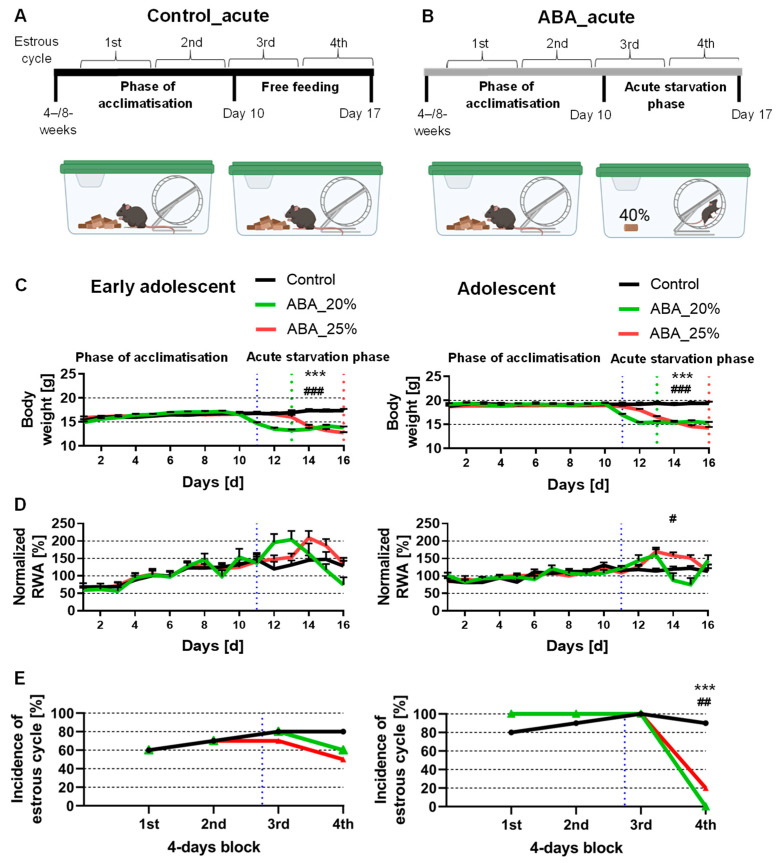
Acute starvation in ABA mice does not combine both AN-related symptoms hyperactivity and amenorrhea. (**A**,**B**) A schematic experimental set-up of acute starvation with early adolescent (4 weeks old) and adolescent (8 weeks old) mice is presented. Created with BioRender.com. (**C**) The body weight, (**D**) standardized running wheel activity (RWA) measured with sensors (the average of the first 10 days was set to 100%), and (**E**) incidence of estrous cycle determined via histological analysis of vaginal smears were measured daily at 1 PM. The blue dotted lines show the beginning of the acute starvation phase. The green dotted line represents when ABA mice reached a 20% weight reduction and the red line represents 25% weight loss achievement. The incidence of mice with an estrous cycle within 4-day blocks is presented as the normal duration of a cycle lasting 4 days. (**C**,**D**) Two-way ANOVA with repeated measurements was used to compare the body weight and RWA between ABA and control mice for the duration of both phases (phase of acclimatization and acute starvation phase). * Control vs. 20% ABA, *** *p* ≤ 0.001, # Control vs. 25% ABA, # *p* ≤ 0.05, ## *p* ≤ 0.01, ### *p* ≤ 0.001. (**E**) Chi-square tests were used to compare the incidence of mice with an estrous cycle between ABA and control mice.

**Figure 2:**
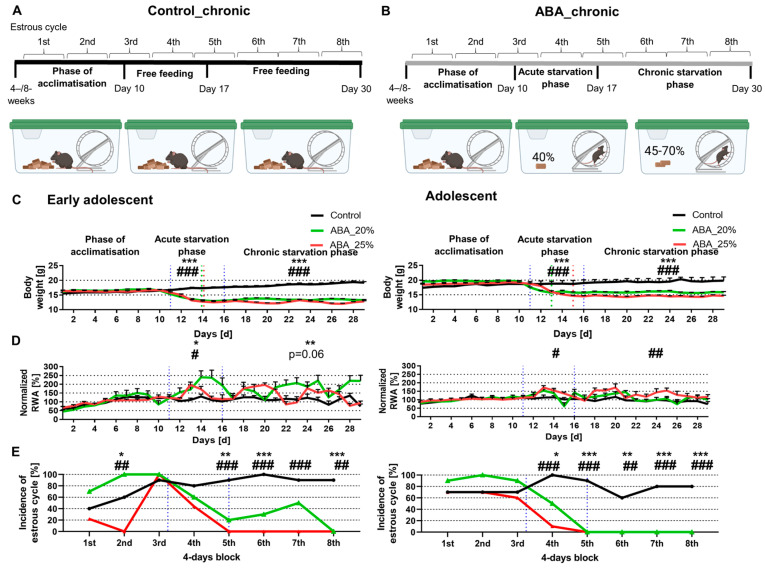
Chronic starvation in ABA mice leads to hyperactivity and amenorrhea, except the adolescent cohort with 20% weight reduction. (**A**,**B**) The schematic structure of chronic starvation with early adolescent and adolescent control and ABA mice is displayed. Body weight, running wheel activity (RWA), and estrous cycle were analyzed at 1 PM, with the cycle blocks of 4 days shown in the chronological course of the ABA model. Created with BioRender.com. (**C**) The body weight, (**D**) standardized RWA (the average of the first 10 days was set to 100%), and (**E**) incidence of mice with estrous in percent during chronic starvation in early adolescent (left) and adolescent mice (right) are shown. Blue dotted lines represent the start of the acute or chronic starvation phase, while green/red lines represent that the ABA mice achieved a 20%/25% weight reduction. (**C**,**D**) Two-way ANOVA with repeated measurements for the duration of both phases (phase of acclimatization, acute starvation phase or chronic starvation phase), * Control vs. 20% ABA, * *p* ≤ 0.05, ** *p* ≤ 0.01, *** *p* ≤ 0.001, # Control vs. 25% ABA, # *p* ≤ 0.05, ## *p* ≤ 0.01, ### *p* ≤ 0.001. (**E**) The incidence of mice with estrous cycles was analyzed using chi-square tests comparing ABA and control mice.

The structure of the ABA model was previously described by Frintrop et al. [15]. All animals were single-housed in a cage with 24 h running wheel access during the whole experiment. At the beginning of the experiment, a ten-day acclimatization phase was included with ad libitum water and food. The mice were fed with normal chow food from Ssniff (Soest, Germany). The mice were then randomly assigned to different groups. Daily at 1 PM, the following parameters were measured: body weight, menstrual cycle, and food intake. During starvation phases, the feeding of the mice was included at this time point. Additionally, we analyzed the locomotor activity, specifically the running wheel activity (RWA), using running wheel sensors with automatic revolution detection, measured hourly. The acute starvation phase included the mice receiving 40% of the baseline food intake calculated with the feeding amount of the acclimatization phase until a 20 or 25% body weight reduction was reached (Control_acute_4W: *n* = 10; ABA_acute_4W20%: *n* = 10; ABA_acute_4W25%: *n* = 10; Control_acute_8W: *n* = 10; ABA_acute_8W20%: *n* = 10; ABA_acute_8W25%: *n* = 10). To determine the baseline food intake, the daily food intake during the acclimatization phase was averaged and 40% of this average amount was calculated. After reaching their target weight, the food intake was adjusted daily to preserve the 20 or 25% weight reduction. After acute starvation for one week, the chronic starvation phase was implemented, in which the mice maintained their target weight for another two weeks (Control_chronic_4W: *n* = 10; ABA_chronic_4W20%: *n* = 10; ABA_chronic_4W25%: *n* = 9; Control_chronic_8W: *n* = 10; ABA_chronic_8W20%: *n* = 10; ABA_chronic_8W25%: *n* = 10). The ABA animals had unlimited access to the reduced amount of food during the acute and chronic starvation phase. In parallel, the control mice were housed with ad libitum food for the whole experiment. Figure 1A,B and Figure 2A,B present a schematic structure of the ABA paradigm.

### 2.3. Locomotor Activity Determination

Every cage was equipped with a running wheel (STARR Life Science Corp., Oakmont, PA, USA). We investigated the RWA with an activity software (VitalView Activity 1.4, STARR Life Science Corp.). Technically, the number of revolutions per hour was analyzed with a magnetic counter.

### 2.4. Estrous Cycle Determination

The evaluation of the menstrual cycle was implemented as defined in a previous study [15]. Briefly, vaginal smears were incubated in a 10% Giemsa solution (Giemsa stock solution, ROTH T862.1, Karlsruhe, Germany). The vaginal smears were classified into estrous (fertile phase), metestrous, diestrous, and proestrous phases. The regular cycle of mice lasts 4 days, and thus, no fertile phase within 4 days was defined as amenorrhea. The incidence of mice with a fertile phase in 4-day blocks is presented in Figure 1E and Figure 2E.

### 2.5. Brain Volume Measurement

After injecting the mice with ketamine (100 mg/kg) and xylazine (10 mg/kg), transcardial perfusions with phosphate-buffered saline (PBS) followed by 3.7% paraformaldehyde solution (pH 7.4) were performed and the brains were carefully dissected. In the next step, the brains were post-fixed in a 3.7% paraformaldehyde solution for one day, rinsed in tap water, and then cryo-protected by immersion overnight in 10%, 20%, and 30% sucrose in PBS at 4 °C. Then, the brains were embedded in an optimal cutting temperature medium (Sakura, Torrance, CA, USA) and kept at −20 °C. The brains were cut frontally in consecutive series of 40 µm thick sections with a cryostat (Leica CM3050 S, Nussloch, Germany) and then every third slide was thaw-mounted on glass slides for brain volume investigations. Afterward, the sections were stained with Nissl standard protocols. Stained slices were digitized and the areas of interest (cerebral cortex) of every second slice were evaluated manually by tracing with the software ImageJ (1.48v, Wayne Rasband, National Institutes of Health, Bethesda, MD, USA). The analysis was performed by an observer blinded to the different groups. To calculate the volumes of interest, we used the Cavalieri method, which involved multiplying the individual areas by the slice thickness and adding up the results. The mouse brain atlas from Paxinos and Franklin was used to include the whole cerebral cortex volume from Bregma 3.20 to −5.20.

### 2.6. Nestling Test

The nesting activity was measured on day 4 of starvation to evaluate the nestling behavior of mice (Control: *n* = 10, ABA: *n* = 10). A cotton nestlet (5 cm square made from crushed cotton fibers, Zoonlab GmbH, Castrop-Rauxel, Germany) was located in the right back corner of the cage 45 to 60 min before the start of the dark phase. A modified scoring system, originally described by Deacon and colleagues, was used to score the nests at the end of the dark phase ±1 h [21]. The modification implemented a score of six, which was defined as a perfect nest. This perfect nest appears like a cavity, and more than 90% of the circumference of the nest wall is higher than the body height of the coiled-up animal [22].

### 2.7. Measurement of Corticosterone Metabolites and Blood Sugar Levels

To evaluate adrenocortical activity [23], feces that had been left in the cages for one whole day were collected before starvation (pre) and on starvation days 1–3 at 1 PM (Control: *n* = 10, ABA: *n* = 10). Afterwards, feces (200–400 mg) were dried for 4 h at 65 °C and stored at −20 °C until further processing. Then, 50 mg of feces were extracted with 1 mL of 80% methanol for further measurement of fecal corticosterone metabolites using 5α-pregnane-3β,11β,21-triol-20-one enzyme immunoassay as previously described [24,25]. Individual blood samples were collected at the end of the experiment (after acute or chronic starvation) via retro-orbital blood-draw. Blood glucose levels were measured immediately after blood collection using a Contour^®^XT glucose meter (Bayer, Leverkusen, Germany), following the manufacturer’s instructions.

### 2.8. Statistics

The data are shown as means and standard errors of the mean (SEM). To evaluate the acclimatization (days 1–10), acute starvation (days 11–16), and chronic starvation phases (days 17–29), the data for body weight and RWA were averaged. The relative RWA increases during acute and chronic starvation were calculated by normalizing the daily average RWA with the RWA during the habituation phase. The analysis of body weight and RWA between ABA and control mice within each phase of starvation was implemented using two-way ANOVA with repeated measurements. We set the significance level to 5% and used Bonferroni’s test for post hoc assessments between the control and ABA groups. Chi-squared tests were used to evaluate if the estrous phase in every 4-day block was changed between the control and ABA groups. Furthermore, the parameters of cortex volumes, corticosterone metabolites, and blood glucose levels were evaluated with Student’s *t*-tests and the nestling score with Mann–Whitney tests. All statistical evaluations were performed using SPSS version 20 (IBM, Chicago, IL, USA).

## 3. Results

### 3.1. Acute Starvation Does Not Lead to Both AN-Related Symptoms, Id Est, Hyperactivity and Amenorrhea

In the beginning, we investigated whether acute starvation leads to AN-related symptoms. The early adolescent ABA mice with 20% weight reduction reached their target weight on day 13, while the early adolescent ABA mice with 25% weight reduction only reached it on day 16 (see the green dotted line for 20% and the red for 25% weight reduction in Figure 1C, left). In comparison, also in the adolescent ABA mice, the mice with 20% weight reduction reached their target weight three days earlier than the mice with 25% weight reduction (Figure 1C). This observation could potentially be attributed to the presence of a greater proportion of resistant ABA animals in the 25% weight reduction groups. Previous studies have indicated that approximately 50% of ABA animals may exhibit resistance to the ABA protocol [26,27].

Acute starvation induced no change in RWA in both early adolescent ABA groups compared to the control group (Figure 1D, left). Additionally, adolescent ABA mice with 20% weight reduction also demonstrated no significant difference in RWA (Figure 1D, right). In contrast, adolescent ABA mice with 25% weight reduction significantly increased their RWA during the acute starvation phase (Control_acute_4W: 117.32% ± 6.46 vs. ABA_acute_4W25%: 138.12% ± 4.5, *p* = 0.02).

After all 4-day blocks, no difference was detected when analyzing the incidence of the estrous cycle between control and early adolescent ABA mice (Figure 1E, left). After the first three blocks, also, no difference was detected when analyzing the incidence of the estrous cycle between control and adolescent ABA mice (Figure 1E, right). In contrast, after the fourth block, a regular estrous cycle was detected in none of the adolescent ABA mice with 20% weight reduction, and in 20% of the adolescent ABA mice with 25% weight reduction (chi-square test, Control_acute_8W vs. ABA_acute_8W20%: χ(df) = 1, χ^2^ = 16.36, *p* = 0.0001; Control_acute_8W vs. ABA_ acute_8W25%: χ(df) = 1, χ^2^ = 9.9, *p* = 0.002). To summarize, acute starvation did not lead to either AN-related symptoms in one cohort, suggesting that acute starvation is not sufficient to mimic core AN-related symptoms.

### 3.2. Chronic Starvation in Early Adolescent Mice Induces Hyperactivity and Amenorrhea

In the next step, we analyzed whether chronic starvation induced AN-related symptoms (Figure 2). Chronic starvation comprised an acute starvation phase (days 11–16) followed by a chronic starvation phase (days 17–29). The early adolescent ABA mice with 20% and 25% weight reduction reached their target weight on day 14 (see green/red dotted lines in Figure 2C, left). Hyperactivity was defined as an increase in RWA during the acute and/or chronic starvation phase. In comparison, the adolescent ABA mice with 20% weight reduction reached their target weight two days earlier than those with a 25% weight reduction.

In comparison with the first experiment (Figure 1), acute starvation led to a significant increase in RWA in both early adolescent ABA groups (Control_chronic_4W: 113.68% ± 10.53 vs. ABA_chronic_4W20%: 185.83% ± 25.88, *p* = 0.02; Control_chronic_4W: 113.68% ± 10.53 vs. ABA_chronic_4W25%: 141.38% ± 4.56, *p* = 0.04, Figure 2D, left). Furthermore, chronic starvation led to a significant increase in RWA in early adolescent ABA mice with 20% weight reduction, while only a trend was shown in early adolescent ABA mice with 25% weight reduction (Control_chronic_4W: 112.21% ± 0.81 vs. ABA_chronic_4W20%: 176.37% ± 21.22, *p* = 0.01; Control_chronic_4W: 112.21% ± 0.81 vs. ABA_chronic_4W25%: 135.66% ± 3.77, *p* = 0.06). In comparison, the RWA of the adolescent ABA mice with 20% weight reduction was not altered compared to the control mice (Figure 2D, right). In contrast, during the acute and chronic starvation phases, the RWA of ABA mice with 25% weight reduction was increased (acute starvation: Control_chronic_8W: 108.78% ± 10.97 vs. ABA_chronic_8W25%: 134.65% ± 5.1, *p* = 0.05, chronic starvation: Control_chronic_8W: 95.19% ± 11.08 vs. ABA_chronic_8W25%: 131.79% ± 5.68, *p* = 0.01, Figure 2D, right).

In terms of the estrous cycle, after the first, third, and fourth blocks, no difference was measured between control and early adolescent ABA mice (Figure 2E, left). After the fifth, sixth, and eighth blocks, a difference in estrous cycle was detected in early adolescent ABA mice with 20% weight reduction. In comparison, after these blocks, a regular estrous cycle was not detected in any of the early adolescent ABA mice with 25% weight reduction (chi-square test, exemplary for block 8: Control_chronic_4W vs. ABA_chronic_4W20%: χ(df) = 1, χ^2^ = 16.36, *p* = 0.0001, Control_chronic_4W vs. ABA_chronic_4W25%: χ(df) = 1, χ^2^ = 15.4, *p* = 0.0001).

In terms of adolescent mice, after the first three blocks, no difference was measured between control and ABA mice (Figure 2E, right). After the fifth, sixth, seventh, and eighth blocks, a regular estrous cycle was detected in none of the adolescent ABA mice with 20% and 25% weight reduction, indicating that chronic starvation induced amenorrhea (chi-square test, exemplary for block 8: Control_chronic_8W vs. ABA_chronic_8W20%: χ(df) = 1, χ^2^ = 10.77, *p* = 0.001, Control_chronic _8W vs. ABA_chronic_8W25%: χ(df) = 1, χ^2^ = 10.77, *p* = 0.001). In summary, chronic starvation in ABA mice induced the AN-related symptoms of hyperactivity and amenorrhea except in the adolescent ABA mice with 20% weight reduction.

### 3.3. Chronic Starvation with 25% Weight Reduction Induces a Brain Atrophy

**Figure 3:**
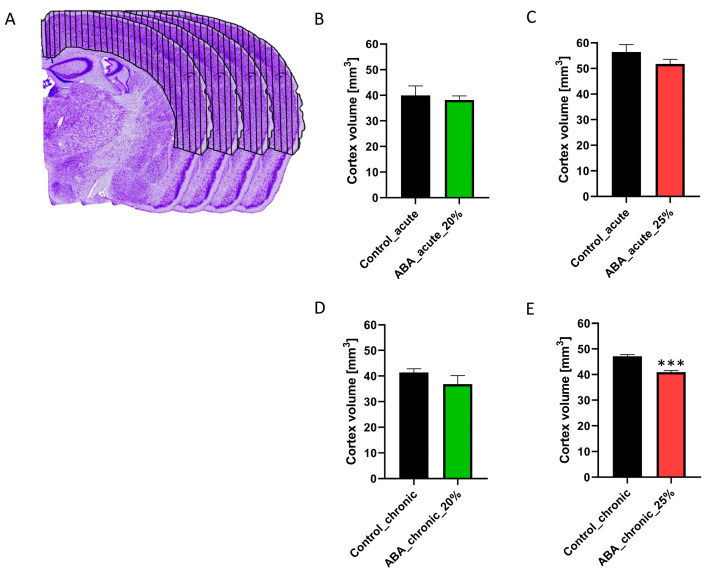
Chronic starvation with 25% weight reduction leads to a reduction in cerebral cortex volume. (**A**–**C**) Representative images of cerebral cortex tracing in Nissl-stained brain sections are presented. Acute starvation did not change the cerebral cortex volume compared to that of control mice. (**D**) Chronic starvation with 20% weight reduction did not induce a change in cerebral cortex volume when comparing control and ABA mice. (**E**) Chronically starved ABA mice with 25% weight reduction revealed a significant reduction in cerebral cortex volume. Two-sided Student’s *t*-test, *** *p* ≤ 0.001.

Next, we aimed to investigate whether starvation leads to brain volume atrophy, estimated via the determination of the cerebral cortex volume by histology (Figure 3).

The findings for cerebral cortex volume were independent of the age of the mice, so we combined the early adolescent and adolescent control and ABA groups. Acute starvation did not lead to a change in cerebral cortex volume between control and ABA mice, regardless of the extent of weight reduction or length of starvation. Furthermore, chronic starvation with 20% weight reduction also did not induce a change in this volume. In contrast, the cortex volume in chronically starved ABA mice with 25% weight reduction was decreased (Control_chronic: 47.71 mm^3^ ± 0.65 vs. ABA_chronic_4W25%: 40.91 mm^3^ ± 0.58, *p* = 0.000002). In summary, only chronic starvation with a 25% weight reduction induced brain atrophy.

### 3.4. Starvation Leads to a Change in Nestling Behavior, an Increase in Corticosterone Metabolites, and a Decrease in Blood Glucose Levels

**Figure 4:**
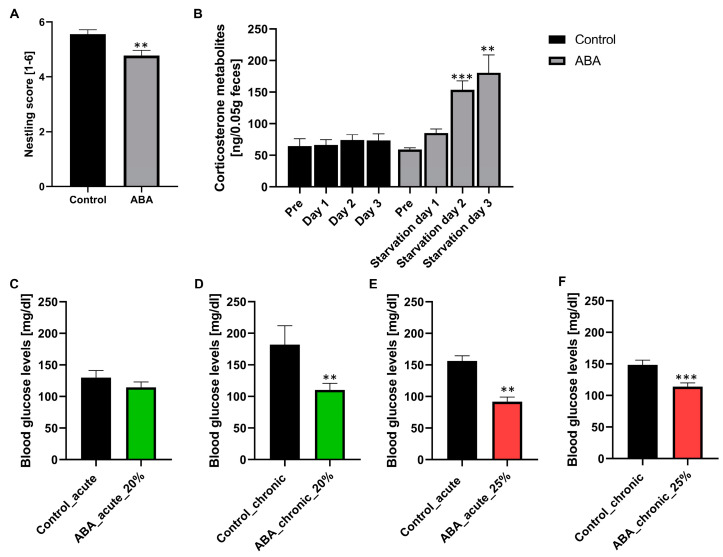
Starvation leads to changes in nestling behavior, an increase in corticosterone metabolites, and decreased blood glucose levels. (**A**) The nestling test was performed on starvation day 4 (Control: *n* = 10, ABA: *n* = 10). Furthermore, the nestling score of ABA mice was altered in comparison to the corresponding control. (**B**) The corticosterone metabolites were analyzed on the day prior to starvation (pre) and starvation days 1–3 (Control: *n* = 10, ABA: *n* = 10). On starvation days 2 and 3, these metabolites were significantly increased in ABA mice. (**C**) Blood glucose levels from each mouse were determined at the end of the experiment. Acute starvation and 20% weight reduction did not induce a change in blood glucose levels. (**D**–**F**) Blood glucose levels were decreased in ABA mice after acute starvation with 25% weight reduction and chronic starvation in comparison to the corresponding control. (**A**) Mann–Whitney test, ** *p* ≤ 0.01. (**B**–**F**) Two-sided Student’s *t*-test, ** *p* ≤ 0.01, *** *p* ≤ 0.001.

To analyze the influence of starvation on stress behavior and the HPA axis, we performed a nestling test on day 4 of starvation and measured the corticosterone metabolites in feces samples the day before starvation (pre) and the first three days of starvation (Figure 4A,B). Furthermore, to analyze the influence of starvation on energy metabolism, blood glucose levels were measured after acute and chronic starvation (Figure 4C–F).

After starvation, the nestling score was significantly reduced in ABA mice compared with control mice (Control: 5.6 ± 0.2 vs. ABA: 4.8 ± 0.33, *p* = 0.01). An average score of five was revealed in ABA mice, representing almost perfect nests, indicating that these mice can and are still motivated to build their nests. Furthermore, the corticosterone metabolites in ABA mice at pre- and starvation day 1 were not altered in comparison to those in control mice. In comparison, these metabolites in ABA mice were significantly increased at starvation days 2 and 3 (starvation day 2: Control: 73.82 ± 8.84 vs. ABA: 153.65 ± 14.01, *p* = 0.0001, starvation day 3: Control: 73.16 ± 10.93 vs. ABA: 180.73 ± 28.06, *p* = 0.002). To summarize, starvation induced a moderate level of stress, evidenced by a change in nestling behavior and an increase in corticosterone metabolites. Furthermore, the blood glucose levels in ABA mice with acute starvation and 20% weight reduction were not altered in comparison to those of control mice. In contrast, the blood glucose levels in chronically starved mice with 20% weight reduction decreased significantly in comparison to those of control mice (Control_chronic: 182.16 mg/dl ± 29.55 vs. ABA_chronic_20%: 110.34 mg/dl ± 10.11, *p* = 0.01). In ABA mice with 25% weight reduction, the blood glucose levels in both acutely and chronically starved mice decreased (Control_acute: 156.40 mg/dl ± 7.94 vs. ABA_acute_25%: 91.75 mg/dl ± 7.57, *p* = 0.00001; Control_chronic: 148.40 mg/dl ± 7.62 vs. ABA_chronic_25%: 113.89 mg/dl ± 5.78, *p* = 0.001).

## 4. Discussion

AN is a serious mental health disorder characterized by a persistent restriction of food intake, a fear of gaining weight or becoming overweight, and a distorted body image. It is accompanied by severe emaciation, extensive locomotor activity, and amenorrhea. In this study, we established a modified ABA model in mice analyzing the parameters (i) extent of body weight reduction (20 vs. 25%), (ii) age of the animals (early adolescent vs. adolescent), and (iii) length of starvation (acute vs. chronic starvation) to enable functional studies. To validate this establishment, the parameters hyperactivity, amenorrhea, and brain atrophy were used.

By comparing the two weight reduction extents of 20% and 25%, it seemed that the 25% weight reduction more closely resembled the behavioral changes seen in AN, particularly the symptoms of amenorrhea and brain atrophy (Table 1). The chronically starved early adolescent ABA mice were more suitable for mimicking AN-related behavioral changes compared with the adolescent ABA mice, as only the early adolescent ABA groups developed hyperactivity. The analysis of the length of starvation showed that acute starvation did not lead to both AN-related symptoms of hyperactivity and amenorrhea in one cohort. In contrast, chronic starvation led to both AN-related symptoms in all cohorts, except for the adolescent ABA mice with a 20% weight reduction. During chronic starvation, the symptom of amenorrhea was more pronounced, which suggests that it may be a secondary dysfunction of the hypothalamus.

Furthermore, the study also analyzed the impact of starvation on brain volume through post-mortem sections, which showed that the volume of the cerebral cortex was only reduced in chronically starved ABA mice with a 25% weight reduction. Additionally, our model also demonstrated an increase in food-anticipatory behavior and changes in circadian-rhythm related activity [28], which are consistent with previous studies using the original ABA model [26,29].

In order to assess whether the starvation protocol had an impact on the mice’s well-being, we evaluated stress-related behavior through two measures: (i) nest-building activity and (ii) levels of corticosterone. We showed that the mice in the ABA group were still able to build an almost perfect nest, indicating that their well-being was not considerably affected by starvation. However, the investigation of the HPA axis showed a moderate increase in corticosterone metabolites in ABA mice on starvation days 2 and 3, which is consistent with previous findings [11,12,13]. It would be interesting to investigate if these metabolites decrease after longer periods of starvation in future studies. Initial findings on energy metabolism revealed that ABA mice with 25% weight reduction demonstrated a decrease in blood glucose levels, indicating depletion of energy stores due to starvation. To gain a deeper understanding of how energy metabolism is affected by starvation, additional planned analyses will investigate the levels of liver glycogen and energy expenditure measured by an open flow indirect calorimetry apparatus [30]. A previous study revealed that the ABA model exhibited hypothermia and an increased browning of adipose tissue, which suggests that starvation leads to alterations in thermogenesis due to changed energy metabolism [31].

One advantage of our modified ABA model is that it is well-suited to mimic the somatic effects of chronic starvation without high distress, loss of animals, or unnecessary suffering. While the mortality rate was increased in the original ABA model [10], none of the ABA mice had to be sacrificed due to starvation in our modified version. Another advantage is that our model allows more precise and controlled manipulation of body weight, leading to more consistent and reliable findings in experimental studies. In addition, different lengths of starvation can be investigated with our model. Another advantage of the ABA model is its usefulness to test drugs such as olanzapine, ketamine, dronabinol, psilocybin, or leptin, which are considered promising candidates for medical interventions in AN, despite not being fully approved for such use [32]. Treating ABA mice with the atypical antipsychotic olanzapine [33,34,35] or ketamine [36] enhanced the survival of ABA animals. Treatment with dronabinol, Δ^9^-tetrahydrocannabinol (THC), reduced survival of these animals; however, food intake was higher in survivors [29]. To the best of our knowledge, the ABA model has not been utilized to test psilocybin, an alkaloid found in certain species of mushrooms [37]. Exner et al. showed that the implication of the satiety hormone leptin reduced hyperactivity in food-restricted rats [38], whereas another study revealed that leptin treatment in ABA rats was not effective [39]. In future studies, one control group without a running wheel and one food-restricted group without a running wheel will be essential to distinguish starvation-induced effects of activity from food restriction. The social isolation of mice is one disadvantage of the applied model, as this might induce stress in the animals; however, for investigating RWA in ABA mice, housing in single cages is necessary. This factor is counterbalanced, as we housed the control mice under the same conditions.

To summarize, hyperactivity was present in all chronically starved ABA mice except the adolescent cohort with 20% weight reduction, while amenorrhea was shown in all chronically starved groups. Further, only chronically starved ABA mice with 25% weight reduction showed brain atrophy. Therefore, to most closely mimic AN-related symptoms, we recommend (i) 25% body weight reduction, (ii) early adolescent or adolescent mice, and (iii) chronic starvation. Our previous studies revealed that GFAP^+^ astrocyte density in chronic ABA rats was reduced in the corpus callosum and cerebral cortex [16,40,41]. By using this new modified murine ABA model in combination with transgenic or knockout mice with specific mutations in astrocyte-related genes, the role of astrocytes and their potential dysfunctions will be analyzed in the near future.

## 5. Conclusions

To conclude, the advantages of modified chronic ABA in mice are that it is easily practicable and decreases harm. Furthermore, it mimics various main hallmarks of human AN in a murine model. Additionally, chronic starvation can be investigated in a controlled manner as an important tool in analyzing the underlying pathophysiology of AN. 

## Figures and Tables

**Table 1:** The parameters of hyperactivity (during acute and/or chronic starvation phase), amenorrhea, and brain atrophy by determining the cerebral cortex volume in different animal groups are summarized. Plus indicates the presence of the different parameters, minus displays the absence.

Animal Group	Hyperactivity	Amenorrhea	Brain Atrophy
20% weight reduction; 4 weeks old mice; acute starvation	-	-	-
25% weight reduction; 4 weeks old mice; acute starvation	-	-	-
20% weight reduction; 8 weeks old mice; acute starvation	-	+	-
25% weight reduction; 8 weeks old mice; acute starvation	+	-	-
20% weight reduction; 4 weeks old mice; chronic starvation	+	+	-
25% weight reduction; 4 weeks old mice; chronic starvation	+	+	+
20% weight reduction; 8 weeks old mice; chronic starvation	-	+	-
25% weight reduction; 8 weeks old mice; chronic starvation	+	+	+

## Data Availability

The data that support the findings of this study are available from the corresponding author upon reasonable request.
